# Post-transcriptional repression of CFP-1 expands the regulatory repertoire of LIN-41/TRIM71

**DOI:** 10.1093/nar/gkad729

**Published:** 2023-09-06

**Authors:** Pooja Kumari, Lars Harald Thuestad, Rafal Ciosk

**Affiliations:** Department of Biosciences, University of Oslo, Oslo 0316, Norway; Department of Biosciences, University of Oslo, Oslo 0316, Norway; Department of Biosciences, University of Oslo, Oslo 0316, Norway

## Abstract

The *Caenorhabditis elegans* LIN-41/TRIM71 is a well-studied example of a versatile regulator of mRNA fate, which plays different biological functions involving distinct post-transcriptional mechanisms. In the soma, LIN-41 determines the timing of developmental transitions between larval stages. The somatic LIN-41 recognizes specific mRNAs via LREs (LIN-41 Recognition Elements) and elicits either mRNA decay or translational repression. In the germline, LIN-41 controls the oocyte-to-embryo transition (OET), although the relevant targets and regulatory mechanisms are poorly understood. The germline LIN-41 was suggested to regulate mRNAs indirectly by associating with another RNA-binding protein. We show here that LIN-41 can also regulate germline mRNAs via the LREs. Through a computational-experimental analysis, we identified the germline mRNAs potentially controlled via LREs and validated one target, the *cfp-1* mRNA, encoding a conserved chromatin modifier. Our analysis suggests that *cfp-1* may be a long-sought target whose LIN-41-mediated regulation during OET facilitates the transcriptional reprogramming underlying the switch from germ- to somatic cell identity.

## Graphical Abstract

**Graphical Abstract:**
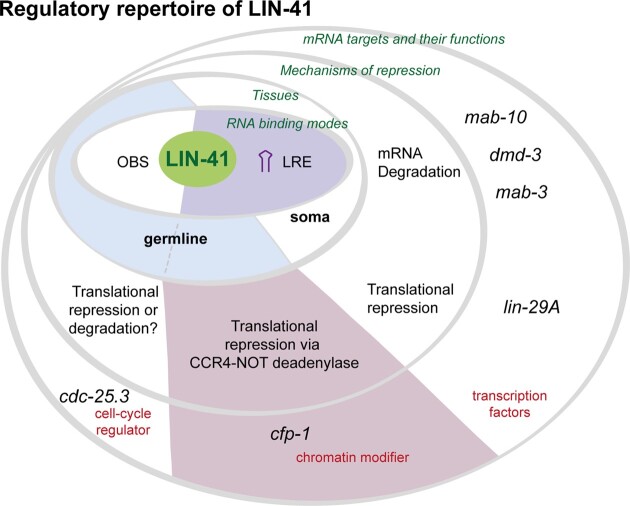

## INTRODUCTION

RNA-binding proteins (RBPs) are versatile regulators of mRNA fate (1). They employ diverse RNA binding domains (RBDs) to recognize specific sequences and/or structural features of RNA (2). The NHL domain, named after the *Caenorhabditis elegans* proteins NCL-1, HT2A and LIN-41, is an RBD found in the conserved TRIM-NHL family of RBPs (3). The NHL repeats form a beta-propeller structure whose top surface can bind RNA in various ways (4). The TRIM-NHL proteins control cell proliferation versus differentiation decisions in many species. Underscoring the importance of RNA regulation, mutations in their NHL domains are associated with numerous diseases, including cancer and neurological disorders (5–8).

The *C. elegans* LIN-41 is a well-characterized TRIM-NHL protein that associates with specific RNAs via tri-loop RNA hairpins called the LIN-41 Response Elements (LREs) (4). LIN-41 is best known as a player in the so-called heterochronic pathway that regulates developmental transitions in the soma (9,10). The somatic LIN-41 regulates target mRNAs via either degradation or translational repression, depending on whether it binds within the 3′ or 5′ untranslated regions (UTRs) of those mRNAs (11). However, the exact molecular mechanisms remain unknown. Additionally, LIN-41 functions in the germline, where it controls various aspects of the OET, including the meiotic progression and reprograming into pluripotency (12–14). In the absence of LIN-41, germ cells abort meiosis, proliferate and abnormally differentiate into somatic cells forming the invertebrate equivalent of a human teratoma (12). The teratomatous differentiation reflects the premature onset of transcriptional reprogramming underlying the so-called embryonic (or zygotic) genome activation, which, during wild-type development, occurs in an early embryo (12). While the function of LIN-41 in the heterochronic pathway is linked to specific mRNAs, the identity of germline targets relevant for the various aspects of OET is less clear. Involvement of LIN-41 in the progression through meiosis I entails the regulation of *cdc-25.3* mRNA, which encodes an activator of the cell cycle kinase CDK-1 (13). However, which target(s) are relevant for the cell fate reprogramming remains unknown.

Intriguingly, the *cdc-25.3* mRNA does not harbor LREs in its UTRs, arguing against the direct regulation by LIN-41. Instead, it has been proposed that LIN-41 is recruited to this and additional germline mRNAs via the association with distinct RBPs called OMA-1 and OMA-2 (collectively referred to as OMA), which bind the *cdc-25.3* mRNA via OMA binding sites (OBSs, UAA/U) (15). The OMA-dependent association of LIN-41 with mRNAs in the germline could reflect fundamental differences in the RNA-binding mechanisms used by LIN-41 in the soma versus germline. Alternatively, both OMA-mediated and LRE-mediated RNA binding could co-exist in the germline to regulate distinct targets with different biological functions. Indeed, we show here that the LRE-mediated regulation is also present in the germline and characterize one target, the *cfp-1* mRNA, which is translationally repressed via LREs. Unlike in the soma, we show that the translational repression (or repression for simplicity) of *cfp-1* involves poly(A) tail shortening via the CCR4–NOT deadenylase. CFP-1 is a conserved CXXC zinc finger protein functioning in different chromatin-modifying complexes (16–18). Our observations implicate it in the transcriptional reprogramming underlying the germline-to-soma transition and suggest that its LIN-41-mediated repression facilitates an orderly OET.

## MATERIALS AND METHODS

### Experimental model: *C. elegans*

*C. elegans* strains were maintained by incubation at 20°C on 2% Nematode Growth Medium (NGM) plates seeded with *Escherichia coli* strain OP50 (19). N2 Bristol strain was used as a wild-type reference of *C. elegans*. All other strains used are listed in Supplementary Table S1, together with their genotype and the strain ID used in the Ciosk lab. PHX3876, PHX5469 and PHX5817 were generated by SunyBiotech. To obtain synchronous worm populations, embryos were extracted from gravid adults with a bleaching solution (30% (v/v) sodium hypochlorite (5% chlorine) reagent (Thermo Fisher Scientific; 419550010), 750 mM KOH) and incubated overnight in the absence of food, at room temperature in M9 buffer (42 mM Na_2_HPO_4_, 22 mM KH_2_PO_4_, 86 mM NaCl, 1 mM MgSO_4_). Arrested L1 larvae were plated on food and incubated at 25°C for the desired number of hours. For RNAi experiments, arrested L1 larvae (unless otherwise specified) were plated on RNAi-inducing NGM agar plates containing *E. coli* HT115 bacteria with plasmids targeting the gene of interest (20).

### Analysis of RIP-seq data and calculation of predicted binding score

The LIN-41 RIP-Seq data and the LRE model used here are described in a previous publication (4). Briefly, LIN-41 binds a tri-loop RNA structure, where the type of nucleotide bases at specific positions in the RNA stem–loop determines the binding strength. Additionally, the binding strength depends on the LRE numbers and whether the LREs are located within 5′ or 3′ UTRs of mRNAs. Thus, to determine predicted binding scores for all mRNAs, the *C. elegans* transcriptome was scanned for putative LREs within 5′ and 3′ UTRs; we used the *C. elegans* transcript annotations from the WormBase version WS259, as described earlier (4). Putative LREs were grouped into four categories (minimal, weak, medium, strong), with twofold threshold steps (0.225, 0.45, 0.9, 1.8) and the predicted binding scores were calculated per mRNA. Finally, the binding scores were plotted against RIP-Seq enrichment values (log_2_) of mRNAs that were enriched 4 folds or more in the RIP-Seq experiment.

### Construction of reporter strains

The germline GFP reporter with the LREs in the 3′ UTR was constructed as described earlier (4). Briefly, using the MultiSite Gateway Technology (Thermo Fisher Scientific) three entry plasmids with the promoter (*mex-5*), gene body (PEST::GFP::H2B) and 3′UTR (*mab-10* 3′UTR LRE sequences within *unc-54* 3′UTR) were combined with the destination vector pCFJ150 (21) resulting in a plasmid containing a promoter, 5′UTR, coding sequence and a 3′UTR. Transgenic animals were obtained by single-copy integration into the *ttTi5605* locus on chromosome II, using the protocol for injection with low DNA concentration (22).

### Microscopy

DIC (Differential Interference Contrast) and fluorescence imaging was carried out with the Zeiss Axio Imager Z1 microscope equipped with a Zeiss AxioCam MRm camera. The images were processed in an identical manner using Fiji and Adobe Illustrator. For whole animal microscopy, animals were mounted on thin agarose pads in 20 mM levamisole. *C. elegans* gonads were dissected from adult animals on glass microscope slides with reaction wells using syringe needles (BD Microlance™ 3). Animal were placed in a droplet of dissecting buffer (1 μl of 10% Tween 20, 12 μl of 100 mM levamisole, 10 μl of 10× M9 and 77 μl of ddH_2_O) (23). Using the two syringe needles, the head of the animal was cut off in a single rapid motion just below the pharynx, resulting in the gonads and gut popping out of the animal. Thereafter, the gut and the body were carefully removed to isolate the gonads for imaging.

### RNA extraction and cDNA synthesis

For whole animals, RNA was purified by the PureLink™ RNA Mini Kit (Invitrogen) using the protocol provided by the supplier. For dissected gonads, RNA was extracted using the protocol described for single worm RNA analysis (24). Briefly, ten dissected gonads per sample per biological replicate were lysed by heating at 65°C in the lysis buffer (5 mM Tris pH 8.0, 0.5% Triton X-100, 0.5% Tween 20, 0.25 mM EDTA and 1 mg/ml proteinase K). Extracted RNA was then treated with dsDNase (Thermo Scientific) to get rid of genomic DNA. cDNA was synthesized using Maxima H Minus First Strand cDNA Synthesis Kit (for dissected gonads) and SuperScript™ IV First-Strand Synthesis System (for whole worms) using protocol from the suppliers. Total and polyA RNA was converted to cDNA by using random hexamers and oligo d(T)_20_ primers, respectively.

### Reverse transcription quantitative PCR (RT-qPCR)

RT-qPCR was performed with the cDNA as template and gene-specific primers (Supplementary Table S2) for amplification using HOT FIREPol® EvaGreen® qPCR Mix Plus (Solis Biodyne) in a LightCycler96 qPCR machine. For qPCR from whole worm RNA, the housekeeping gene *act-1* was used as an internal control to normalize the PCRs for the amount of RNA used in the reverse transcription reaction. Analysis for fold change was performed with the ${2}^{ - \Delta \Delta {C}_{\mathrm{q}}}$ method (25). For qPCR from gonads, relative abundance for different mRNAs was measured using a standard curve (26). The statistical significance of difference between the two conditions was calculated using the unpaired *t-*test. All primers are listed in Supplementary Table S2.

### 3′ Rapid amplification of cDNA ends (RACE)

Total RNA used for the 3′ RACE experiment was extracted from embryos prepared from wild-type animals as described above. The 3′ RACE System from Invitrogen™ (Catalog number: 18373019) was used for cDNA synthesis according to the kit protocol with 2.5 μg total RNA per reaction. To control RNA and primer quality, cDNA was also synthesized using the FIREScript® RT cDNA synthesis MIX with Oligo (dT) primers from Solis BioDyne. PCR reactions were set up using the 5X FIREPol® Master Mix (Solis BioDyne). Each reaction was set up identically using 2 μl cDNA, 1 μl gene specific primer (10 μM) and 1 μl universal adapter primer provided from the Invitrogen™ 3′ RACE System kit in a total reaction volume of 50 μl. The gene specific *cfp-1* primers used were: *cfp-1* F1 and *cfp-1* F2 (Supplementary Table S2). For the Oligo (dT) primed cDNA controls *cfp-1* R1 or *cfp-1* R2 (Supplementary Table S2) were used instead of the universal adapter primer from the RACE kit. PCR products were separated on a 1.5% agarose TAE (Tris–acetate–EDTA) gel and individual bands were cut out, purified and sequenced.

### Quantification of the *cfp-1* 3′UTR reporter GFP and the CFP-1::mCherry-myc fusion protein

To estimate differences in the expression of *cfp-1* 3′UTR GFP reporters (with and without LRE-disrupting mutations), we measured GFP abundance based on mean fluorescence pixel intensity. The intensities were measured within oocyte nuclei, except in the –1 oocyte where LIN-41 is being degraded (27). To normalize the data, the values were subsequently divided by the mean pixel intensity from arbitrarily selected areas in the distal gonad where LIN-41 is absent. The measurements were taken using Fiji. The means of the normalized oocyte intensities per animal were calculated for 7–10 animals for each reporter strain. The levels of CFP-1::mCherry-Myc protein, in wild-type and *lin-41(tn1487ts)* backgrounds, were quantified based on the mCherry fluorescence, as explained above for the GFP. The data was plotted as a boxplot using R, and the statistical significance of difference between the two strains was calculated using the unpaired two-samples Wilcoxon test.

### Quantification of the somatic LRE reporters

To estimate differences in the expression of the somatic LRE GFP reporters between mock and different RNAi-treated animals, we measured GFP abundance based on mean fluorescence pixel intensity, which was measured for 6–12 hypodermal cells per animal. To normalize the data, background mean fluorescence pixel intensity was measured in areas next to the hypodermal cells and this signal was subsequently subtracted from the reporter signal. The measurements were taken using Fiji from at least five animals for each condition, resulting in the measurement of at least 30 cells per condition. The data was plotted as a boxplot using R, and the statistical significance of difference between mock treated and RNAi treated animals was calculated using the unpaired two-samples Wilcoxon test.

## RESULTS

### LIN-41 can regulate mRNAs in the germline via LREs

**Figure 1.**
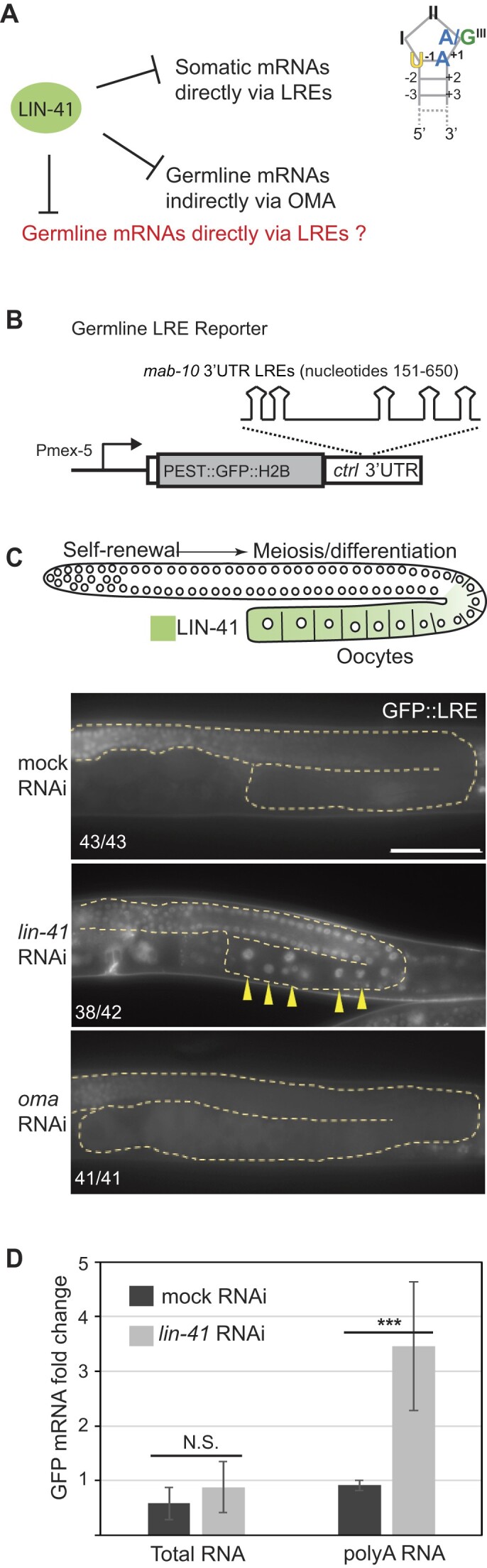
LIN-41 can regulate mRNAs in the germline via LREs. (**A**) Tissue-specific mRNA regulation modes by LIN-41. In the somatic tissue, LIN-41 regulates mRNAs by direct association with the LIN-41 response elements (LREs). LRE is a tri-loop RNA hairpin in which the third nucleotide in the loop is a purine base and the first stem base pair is U–A or C–G. In the germline, LIN-41 is known to regulate some mRNAs by cooperating with another RBP, OMA, which binds RNA via the OMA binding sites (OBS, UAA/U). Whether LIN-41 regulates other mRNAs via the direct binding of LREs is not known. (**B**) Schematic of a germline LRE reporter, wherein GFP fused to PEST and H2B fragments is expressed under the control of the *mex-5* promoter and a synthetic 3′ UTR containing five LREs from the somatic mRNA target (*mab-10*). (**C**) Top: Diagram representing a *C. elegans* gonad, where LIN-41 expression in the proximal gonad in the developing oocytes is shown in green. Lower panels: fluorescent micrographs of gonads from adults expressing the LRE reporter (shown in B). Animals were subjected to mock, *lin-41* or *oma* RNAi. The gonads are outlined with dotted lines. Arrowheads point to examples of nuclei in which the reporter GFP is de-repressed. The numbers indicate how many animals out of total display the presented phenotype. Scale bar = 50 μm. (**D**) RT-qPCR analysis showing relative levels of the GFP reporter mRNA (total versus polyadenylated) in animals treated with mock or *lin-41* RNAi. Error bars represent standard deviation from three biological replicates and *** denotes a *P*-value of <0.05 by an unpaired *t*-test. N.S.= non-significant.

We have previously shown that the NHL domain of LIN-41 binds to target mRNAs in the soma via LREs (Figure 1A and (4)). However, the association of LIN-41 with several germline mRNAs is reported to be indirect, depending on its interaction with the OMA RBPs that bind a distinct RNA motif (Figure 1A and (13,28). To examine whether LRE-mediated mRNA regulation is possible in the germline, we constructed a reporter strain expressing GFP (fused to PEST for rapid turnover and histone H2B to concentrate the signal in the nuclei, facilitating imaging) from a germline promoter (P*mex-5*) and under the control of a 3′ UTR containing LREs from a somatic LIN-41 target (*mab-10*) (Figure 1B). In the wild type, this reporter was expressed in the distal germline but not in the oocytes expressing LIN-41. Consistent with LIN-41-mediated regulation, the reporter was de-repressed upon *lin-41* RNAi but not *oma* RNAi (Figure 1C). Change in the reporter GFP expression could reflect either the degradation or translational repression of the reporter mRNA in wild-type/mock RNAi-ed animals. To distinguish between these two scenarios, we compared the levels of total reporter mRNA in mock versus *lin-41* RNAi-treated animals by RT-qPCR (using random hexamers to synthesize cDNA). We observed no significant difference, arguing against mRNA degradation (Figure 1D). Regulation of the poly(A) tail length is a common mechanism that controls the efficiency of mRNA translation during oogenesis and early embryonic development (29,30). Therefore, we also compared the levels of polyadenylated reporter mRNA by RT-qPCR (using oligo dT primers to synthesize cDNA). We observed enrichment in the poly(A) fraction of reporter mRNA upon *lin-41* RNAi (Figure 1D). This suggests either the retention or extension of the poly(A) tail in the absence of LIN-41. Concluding, LIN-41 appears to be capable of translationally repressing mRNA via LREs also in the germline, through a mechanism that may involve cytoplasmic deadenylation. Therefore, we set out to identify the physiological germline targets of LIN-41 regulated by this mechanism.

### The LRE-containing germline mRNAs include the cfp-1 mRNA

**Figure 2.**
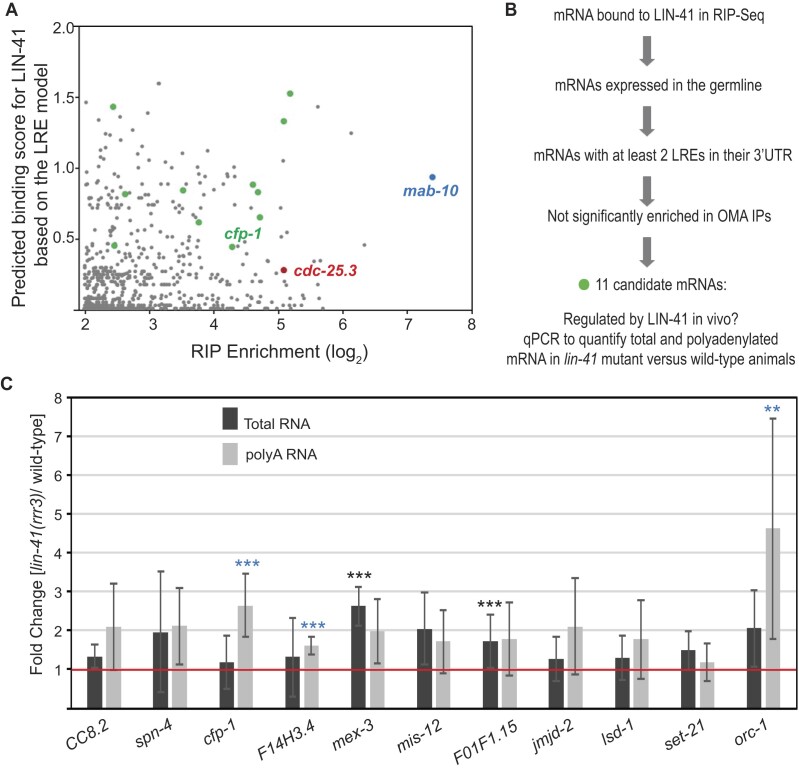
Identification of germline mRNAs potentially regulated via LREs. (**A**) Scatterplot showing (lack of) correlation between mRNAs enriched in LIN-41 RIP (log_2_ scale) and mRNAs predicted to bind LIN-41 via LREs. The *mab-10* mRNA, a somatic LIN-41 target containing LREs is shown in cyan, and *cdc-25.3*, a germline mRNA lacking LREs but regulated by LIN-41 is shown in red. Green dots represent putative LIN-41 germline targets containing LREs identified as described in (B). (**B**) A discovery pipeline used to identify potential LIN-41 mRNA targets in the germline regulated via LREs. (**C**) RT-qPCR analysis comparing relative levels of total and polyadenylated candidate mRNAs between wild-type and *lin-41(rrr3)* mutant animals. The bars represent the ratio of expression levels in *lin-41(rrr3)* mutants to wild-type animals and are therefore compared to 1 as indicated by the red line. Error bars represent standard deviation from three biological replicates. *** denotes a *P*-value of <0.05 and ** a *P*-value of <0.01, by unpaired *t*-test. Blue colored *** indicate significant change in the levels of polyadenylated mRNA whereas black colored *** indicate significant change in the levels of total mRNA.

Our starting point were over 600 mRNAs that co-purify with LIN-41; they were identified by RNA immunoprecipitation, followed by RNA sequencing (RIP-seq) (4). These included the functionally relevant LIN-41 targets in the soma (*mab-10, mab-3, lin-29A* and *dmd-*3) and in the germline (*cdc-25*.3) (11,13). Using the LRE model, we scanned through the predicted secondary structures of mRNAs enriched in the LIN-41 IP and calculated the predicted LIN-41 binding scores. Plotting the predicted binding scores against the RIP-Seq enrichment revealed no correlation between the two, suggesting that LIN-41 also associates with mRNAs lacking LREs (Figure 2A). One example is the known germline target, *cdc-25.3* mRNA, whose regulation does not involve LREs. Nonetheless, the somatic mRNAs regulated by LIN-41 via LREs (e.g. *mab-10*) had high binding scores. Therefore, to identify the putative endogenous germline targets of LIN-41 repressed via the LREs, we applied the following discovery pipeline (Figure 2B). First, we selected only germline-expressed mRNAs. Second, we considered mRNAs harboring at least two LREs in their UTRs, as LRE-mediated regulation requires at least two LREs (our observations and (31)). Third, in order to exclude OMA-dependent binding, we removed mRNAs significantly enriched in OMA RIP (28). This analysis resulted in 11 candidate mRNAs, which contained LREs in the 3′ UTR but none in the 5′ UTRs (green dots, Figure 2A). In order to check if these candidate mRNAs display LIN-41-dependent changes in polyadenylation, as observed for the LRE reporter (Figure 1D), we compared the levels of total and poly-adenylated candidate mRNAs (by RT-qPCR) between wild-type and *lin-41(rrr3)* null mutant animals (Figure 2C). Three candidates were enriched in the poly(A) but not total RNA fraction in *lin-41(rrr3)* mutants (Figure 2C). Amongst these candidates, o*rc-1* mRNA has been previously reported to be regulated by LIN-41 (28). ORC-1 is an essential component of the origin recognition complex required for DNA replication and may have a function in cell-cycle and meiotic maturation. However, its relationship to *lin-41* or *oma* phenotypes is not reported. *F14H3.4* codes for a yet uncharacterized protein with a coiled-coiled domain and two disordered regions. By contrast, CFP-1 is the homolog of mammalian CXXC1 (CFP1) and yeast SPP1, which are chromatin-targeting subunits of the COMPASS (Complex Proteins Associated with Set1) histone methyltransferase complex (32). Thus, we decided to pursue it further.

### LIN-41 regulates cfp-1 via LREs

**Figure 3.**
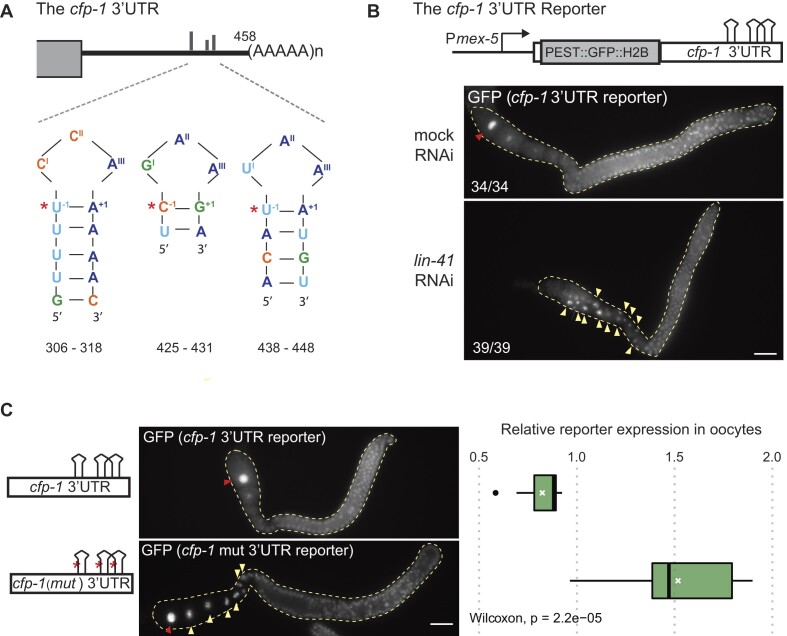
The *cfp-1* mRNA is a bona fide LIN-41 target. (**A**) Schematic of the *cfp-1* 3′ UTR determined by 3′ RACE. The 3′ UTR is 458 nt-long and contains three predicted LREs at the indicated positions. The red asterisks indicate the nucleotides that are mutated in panel C. (**B**) Top: Diagram representing a germline reporter, wherein GFP fused to PEST and H2B fragments is expressed under the control of *mex-5* promoter and *cfp-1* 3′ UTR. The LREs are shown as stem-loops. Lower panels: Fluorescent micrographs of gonads (outlined) dissected from animals expressing the *cfp-1* 3′ UTR reporter, subjected to mock or *lin-41* RNAi. Oocyte nuclei expressing strong GFP fluorescence are marked with yellow arrowheads. The ovulating (–1) oocyte in the mock-treated gonad is marked with a red arrowhead. The numbers indicate how many animals out of total display the presented phenotype. Scale bar = 25 μm. (**C**) Left: Schematics representing the 3′ UTR variations used in the reporters. The red asterisks indicate the mutant nucleotides marked in panel A. Middle: fluorescent micrographs of gonads (outlined) dissected from animals expressing the GFP reporter under the control of wild-type or mutated *cfp-1* 3′UTR. Oocyte nuclei expressing strong GFP are marked with yellow arrowheads. The ovulating (–1) oocytes are marked with red arrowheads. Scale bar = 25 μm. Right: quantification of pixel intensity illustrating the change in reporter GFP fluorescence, calculated as ratio between fluorescence in the oocyte nuclei versus in the distal gonad (oocyte GFP expression/distal gonad GFP expression). The ovulating (–1) oocytes were not included in this analysis. Mean values are marked by white crosses. The *P*-value was calculated using the unpaired two-sample Wilcoxon test.

To verify LIN-41-mediated regulation of *cfp-1* mRNA, we created a reporter strain (similar to the LRE reporter in Figure 1B) where the expression of GFP is under the control of *cfp-1* 3′ UTR. Two 3′ UTR isoforms were annotated for *cfp-1*, of which one lacks the region containing three predicted LREs (Supplementary Figure S1A, WormBase WBGene00009924#0–9f-10). To experimentally validate these isoform(s), we performed a 3′RACE experiment using total RNA extracted from embryos of wild-type animals (Supplementary Figure S1B). We identified only one 3′ RACE product corresponding to the *cfp-1* 3′ UTR that matched the longer 3′ UTR isoform containing the three predicted LREs (Supplementary Figure S1C–E and Figure 3A). This does not exclude the existence of other isoforms expressed at lower levels and/or other developmental stages. Nonetheless, to evaluate LIN-41-mediated regulation through the identified 3′ UTR isoform, we created the corresponding *cfp-1* 3′ UTR GFP reporter strain and examined the GFP expression in animals subjected to either mock or *lin-41* RNAi. In control animals, we observed a stronger GFP expression in the distal gonad but significantly reduced in the developing oocytes, which increased again in the ovulating (–1) oocyte (Figure 3B). This expression pattern anti-correlates with LIN-41, as LIN-41 is expressed in the developing oocytes except in the ovulating (–1) oocyte, where it is actively degraded via the proteasome (27). In agreement with LIN-41 mediated regulation, the reporter GFP expression was no longer reduced in *lin-41* RNAi-treated gonads (Figure 3B). Finally, to confirm that the *cfp-1* LREs mediate the repression, we created a variant of the *cfp-1* 3′ UTR GFP reporter, where single point mutations disrupted the LRE structures necessary for the association with LIN-41 (Supplementary Figure S2). Consistent with the de-repression of this reporter variant, the GFP signal in the oocytes was significantly increased compared to the wild-type reporter (Figure 3C). Taken together, our observations support the LRE-mediated translational repression of *cfp-1* by LIN-41.

### The *cfp-1* regulation involves the CCR4 NOT deadenylase complex

**Figure 4.**
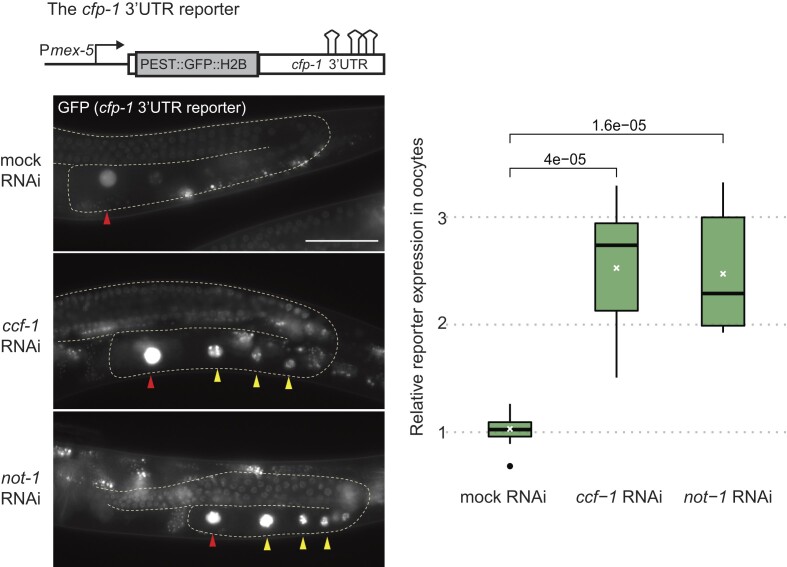
The CCR4–NOT deadenylase complex is required for the translational repression of *cfp-1*. Left: on top is shown a diagram illustrating the *cfp-1* 3′UTR reporter as in Figure 3B. Below are fluorescent micrographs of gonads (outlined) in live animals expressing the *cfp-1* 3′ UTR reporter and subjected to mock, *ccf-1*, or *not-1* RNAi. Yellow arrowheads point to examples of nuclei with strong GFP fluorescence. The –1 ovulating oocytes are indicated by red arrowheads. Scale bar = 50 μm. Right: quantification of pixel intensity illustrating the change in reporter GFP fluorescence, calculated as ratio between fluorescence in the oocyte nuclei versus in the distal gonad (oocyte GFP expression/distal gonad GFP expression). The ovulating (–1) oocytes were not included in this analysis. Mean values are marked by white crosses. The *P*-value was calculated using the unpaired two-sample Wilcoxon test.

Our RT-qPCR experiments suggest that LRE-mediated regulation in the germline involves target mRNA deadenylation (GFP mRNA in Figure 1D and *cfp-1* mRNA in Figure 2C). Purification and identification of LIN-41 partner proteins by immunoprecipitation followed by mass-spectrometry has shown that LIN-41 interacts with the components of CCR4–NOT deadenylase complex (28). Additionally, by systematically analyzing the function of different deadenylases in *C. elegans* germline development, it has been determined that CCR4–NOT complex is the major deadenylase complex in *C. elegans* germ cells and CCF-1 is the main deadenylase (33). To test if the CCR4–NOT deadenylase complex regulates *cfp-1* mRNA, we RNAi-depleted mRNAs encoding the scaffolding protein NOT1 (*ntl-1* in *C. elegans*) and the deadenylase CCF-1 and monitored the expression of the *cfp-1* 3′ UTR GFP reporter. Knocking down these factors resulted in severe developmental defects including in the germline, as observed previously (33). Therefore, we optimized the RNAi-mediated depletion, treating animals from mid-L4 to young adult stage (for 12–14 h), which had no overt impact on oocyte appearance. Such short treatment was sufficient to observe an increase in the expression of the *cfp-1* 3′ UTR GFP reporter upon RNAi against either *not-1* or *ccf-1* (Figure 4). These observations suggest a model, where LRE-associated LIN-41 regulates the associated mRNA by recruiting the CCR4–NOT deadenylase complex. Interestingly, this mechanism appears to be specific to the germline, as the CCR4–NOT complex seems dispensable for the regulation of somatic LIN-41 targets (Supplementary Figure S3). Thus, LIN-41 utilizes different mechanisms to regulate germline versus somatic targets.

### *CFP-1 protein is over-expressed in lin-41* mutant gonads

**Figure 5.**
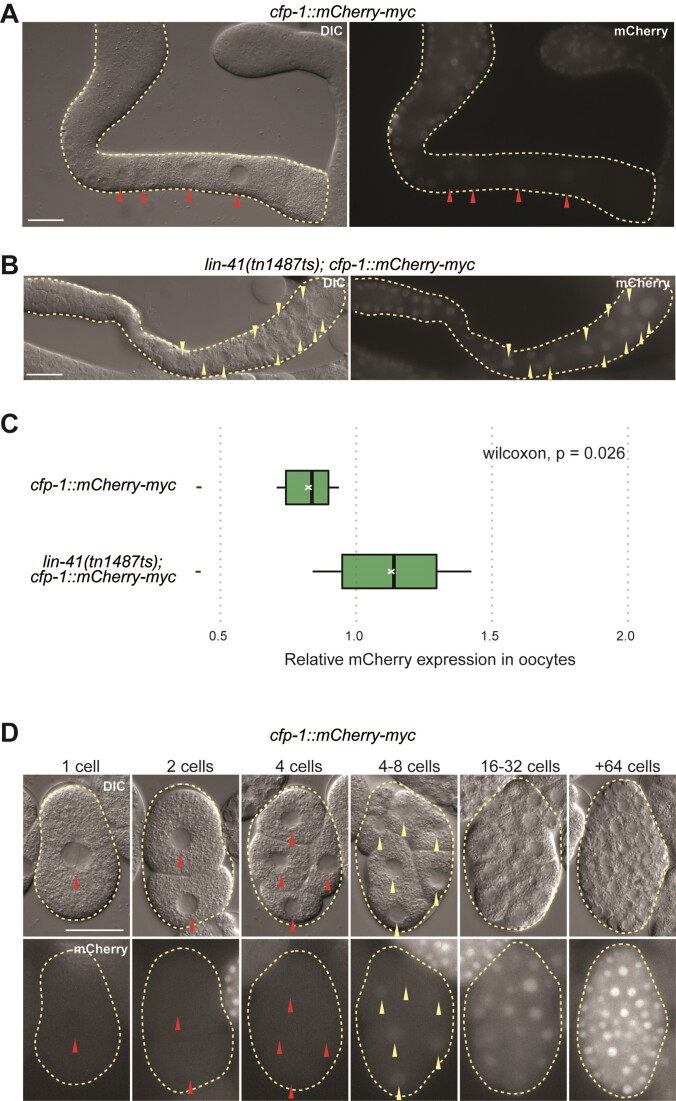
LIN-41 represses CFP-1 expression in the developing oocytes. (**A**–**C**) DIC and fluorescence micrographs from the *cfp-1::mCherry-myc* strain, expressing endogenous CFP-1 fused to mCherry and myc. (A) Gonad dissected from the animal expressing CFP-1::mCherry-Myc. Red arrowheads point to the oocyte nuclei with weak or no mCherry fluorescence. Scale bar = 25 μm. (B) Gonad dissected from the *lin-41(tn1487ts); cfp-1::mCherry-myc* animal. Note the abnormal oocytes in the proximal gonad. Yellow arrowheads point to the nuclei with stronger mCherry fluorescence. Scale bar = 25 μm. (C) Quantification of pixel intensity illustrating the difference in mCherry fluorescence between *cfp-1::mCherry-myc* and *lin-41(tn1487ts); cfp-1::mCherry-myc* animals, calculated as ratio between fluorescence in the oocyte nuclei versus in the distal gonad (oocyte mCherry expression/distal gonad mCherry expression). Mean values are marked by white crosses. The *P*-value was calculated using the unpaired two-sample Wilcoxon test. (**D**) Embryos at the indicated developmental stages. Note that the *C. elegans* embryonic transcription begins around the 4-cell stage. Red arrowheads point to the nuclei with weak or no mCherry fluorescence. Yellow arrowheads point to the nuclei with stronger mCherry fluorescence. Scale bar = 25 μm.

The experiments so far showed that LIN-41 elicits LRE-mediated translational repression of a reporter GFP. To confirm that this mechanism also impacts the levels of CFP-1 protein, we generated a strain expressing endogenous CFP-1 tagged (by CRISPR-Cas9 editing) with mCherry-Myc (*cfp-1(syb3876)*, henceforth *cfp-1::mCherry-myc*). We observed expression of the CFP-1 fusion protein through larval development both in the soma and the germline (Supplementary Figure S4). However, its expression was strongly reduced in the proximal gonad of wild-type animals containing LIN-41 (Figure 5A). To confirm that the levels of CFP-1 depend on LIN-41, we subjected the *cfp-1::mCherry-myc* animals to *lin-41* RNAi (from the L1 to young adult stage). Consistent with the regulation by LIN-41, the expression of CFP-1::mCherry-Myc extended to the proximal gonad in the RNAi-treated animals (Supplementary Figure S5B). To rule out secondary effects due to oocyte malformation, we also examined a *lin-41* temperature sensitive mutant, *lin-41(tn1487ts)*, whose gonads at restrictive temperature still contain oocyte-like cells rather than a teratoma (13). Using this mutant, we also observed the ectopic expression of CFP-1::mCherry-Myc in the oocyte-like cells (Figure 5B, C). Additionally, we examined the CFP-1::mCherry-Myc expression in wild-type embryos. We detected CFP-1 expression starting from the 4–8 cell-stage, with subsequent increase in older embryos (Figure 5D). We noticed a small difference in the expression patterns between the CFP-1 fusion protein and the *cfp-1* 3′UTR GFP reporter (Figure 3B and 5A). While the reporter GFP was visible already in 2 cell-stage embryos, the fusion protein accumulated with some delay (Figure 5D and Supplementary Figure S5A), possibly suggesting regulation by additional mechanisms (see Discussion). Taken together, our results support LIN-41-mediated translational repression of CFP-1 in the proximal gonad.

### CFP-1 promotes the expression of early embryonic genes

**Figure 6.**
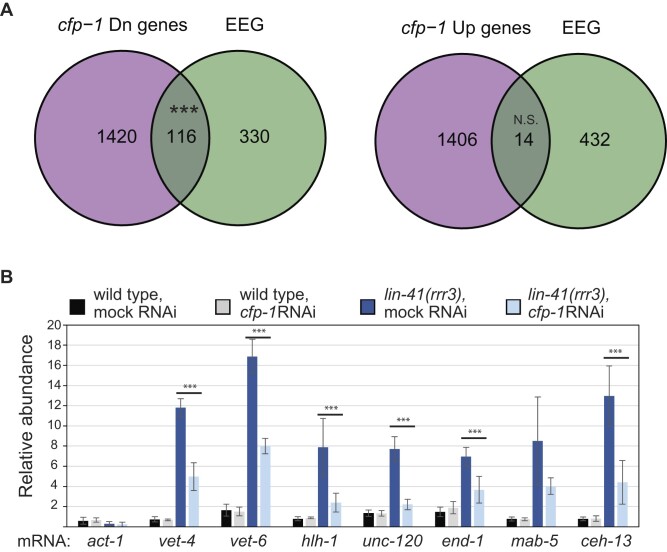
CFP-1 is required for the expression of early embryonic genes in wild-type embryos and *lin-41(-)* mutant gonads. (**A**) Venn diagram showing an overlap between early embryonic genes and differentially expressed genes in the *cfp-1(tm6369)* animals. *** indicates the *P*-value of 1.87e-33, by hypergeometric test. The *cfp-1*(*tm6369*) mutant gene expression data set is taken from Beurton *et al.* and the EEG list is taken from Fassnacht *et al.* Dn = downregulated and Up = upregulated. (**B**) RT-qPCR analysis comparing the abundance of selected mRNAs in the gonads dissected from animals of the indicated genotypes. *act-1* served as a negative control, the remaining mRNAs are normally expressed in embryos. Error bars represent standard deviation from three biological replicates and *** denotes a *P*-value of <0.05, by unpaired *t*-test. Note that, compared with wild type, the levels of embryonic mRNAs were higher in the *lin-41(rrr3)* gonads, but that increase was suppressed upon *cfp-1* RNAi.

CFP-1 is a conserved protein studied from yeast to humans (32). The mammalian CFP1/CXXC1 recruits the SET1/COMPASS to induce the tri-methylation of histone H3 at Lys4 (H3K4me3) at actively transcribed genes. Additionally, recent studies using *C. elegans* show that CFP-1 interacts also with additional chromatin factors, including components of the SIN3/HDAC complex (16,17). Since CFP-1 is absent in the developing oocytes but accumulates in early embryos, we wondered if its abnormal accumulation in *lin-41* mutants could facilitate the transcriptional reprogramming resulting in a teratoma. We first asked whether there is a relationship between CFP-1 and the genes transcribed during embryonic genome activation (26). Specifically, we examined if the early embryonic genes (EEGs) are down or upregulated in *cfp-1* mutants using published transcriptomics data (16). We found a striking enrichment of EEGs among the downregulated but not upregulated genes (Figure 6A). Gene Ontology enrichment analysis showed that EEGs down-regulated in *cfp-1* mutants are functionally related to transcription (dsDNA binding, transcription regulatory region nucleic acid binding, transcription factor activity etc.; Supplementary Figure S6).

We then asked if the ectopic expression of CFP-1 may promote the induction of embryonic genes in *lin-41* gonads. To do that, we dissected gonads from *lin-41(rrr3)* animals subjected to either mock or *cfp-1* RNAi and performed RT-qPCR for specific genes. We selected genes representing different aspects of embryonic differentiation that were previously shown to be abnormally induced in LIN-41-deficient gonads (12). Expectedly, genes representing EEGs (*vet-4*, *vet-6*), somatic lineage specific genes (*hlh-1, unc-120, end-1*) and hox genes (*mab-5, ceh-13*), were all upregulated in *lin-41(rrr3)* mutants compared with wild-type (Figure 6B). However, RNAi-mediated depletion of CFP-1 strongly reduced the levels of most tested transcripts (Figure 6B). Taken together, our experiments suggest that LIN-41-mediated translational repression of *cfp-1* mRNA helps maintain the germ cell fate in the developing oocytes by preventing an untimely onset of embryonic transcription. Importantly, the underlying mechanism of translational repression is distinct from what is reported for other known LIN-41 mRNA targets. Unlike *cdc-25.3*, the repression of *cfp-1* does not require the OMA RBP. Although the repression of *cfp-1* is mediated by LREs, like the repression of somatic targets, the CCR4–NOT deadenylase complex is essential only for the former. Thus, LIN-41 appears to have evolved unique solutions to the regulation of different targets performing distinct biological roles.

## DISCUSSION

**Figure 7.**
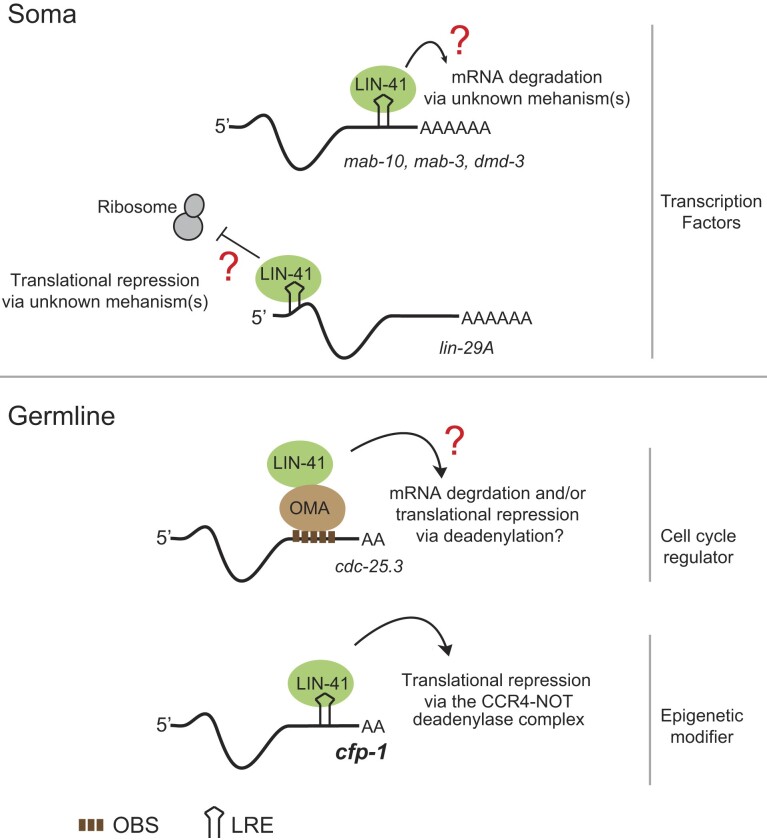
The summary of LIN-41-mediated mRNA regulation Upper panel: in the soma, LIN-41 binds to mRNAs via LREs located in the 3′ or 5′ UTRs. Binding to the 3′ UTRs results in mRNA degradation, whereas binding to the 5′ UTR in translational repression (11). The underlying mechanisms and effectors remain unknown as indicated by the red question mark. The known somatic mRNA targets of LIN-41 encode transcription factors. Lower panel: in the germline, LIN-41 was suggested to bind several mRNAs (including the *cdc-25.3* encoding a cell cycle regulator) indirectly, possibly via the association with the OMA RBP, which binds this and other mRNAs via OMA binding sites (OBSs; consisting of a repetitive UAA/U motif). Whether *cdc-25.3* and/or other indirect LIN-41 targets are regulated via deadenylation is not clear, as indicated by the red question mark (28). Additionally, using the example of *cfp-1* mRNA encoding a chromatin modifier, we showed that LIN-41 regulates germline mRNAs by directly associating with them via LREs. In the case of translational repression *of cfp-1* mRNA, this involves deadenylation via the CCR4–NOT complex.

The *cfp-1* mRNA adds to a growing number of LIN-41 targets regulated by target-specific mechanisms (Figure 7). Previous reports postulated an indirect recruitment of LIN-41 to some germline transcripts, via its association with the OMA RBP. By contrast, our data suggest a direct recruitment of LIN-41 to *cfp-1* through the LREs. Our results suggest that this leads to translational repression involving the CCR4–NOT deadenylase complex. Controlling gene expression through polyadenylation/deadenylation is widespread in germ cells and early embryos (29,30). It was suggested that transcripts associating with LIN-41 are substrates of the GLD-2 polyA polymerase, and both GLD-2 with its co-factors and CCR4–NOT components were identified in the LIN-41 pull-downs (28). Thus, a tug-of-war between cytoplasmic deadenylation and polyadenylation could decide the fate of *cfp-1* mRNA, as was previously suggested for other germline transcripts (34). Interestingly, like LIN-41, also the fly TRIM-NHL protein BRAT utilizes the CCR4–NOT complex to control mRNA translation (35,36). The CCR4–NOT recruitment is mediated by BRAT’s interactions with another RBP, Nanos, which directly associates with the Not1 and Not3 components of the CCR4–NOT complex (37). Whether LIN-41 recruits the CCR4–NOT complex directly or through interacting proteins remains to be tested. Although the transient translational repression of *cfp-1* via deadenylation could largely explain the CFP-1 expression pattern, other mechanisms may contribute. We noticed some differences between the expression patterns of the *cfp-1* 3′ UTR GFP reporter and the endogenous CFP-1 protein. The reporter expression anti-correlates with the abundance of LIN-41. While it is repressed in most oocytes, it is de-repressed in the ovulating (–1) oocytes and early embryos, where LIN-41 is degraded (27). By contrast, the CFP-1 protein is detectable in neither the –1 oocytes nor early embryos. While other explanations remain possible, these observations suggest an additional layer of CFP-1 regulation, possibly involving its proteolysis.

Regardless of the actual mechanism(s), why is CFP-1′s expression controlled? CFP-1 is required for fertility and *cfp-1(tm6369)* mutants have reduced brood size when grown at 25°C, eventually leading to complete sterility within two generations (16,17). Our observations implicate CFP-1 in the expression of early embryonic genes. Whether this function is related to the gonadal or embryonic expression of CFP-1, and whether it is connected to the sterility phenotype, remains to be tested. However, loss of *cfp-1* in the germline results in a phenotype stronger than COMPASS inactivation, most likely reflecting additional functions such as SIN3 recruitment to H3K3me3 enriched promoters (17). It is possible that CFP-1 acts as a hub to coordinate H3K4 tri methylation and HDAC activity. An important question for the future is the relative importance of the CFP-1-containing complexes for the expression of embryonic genes. Similar to CFP-1, the murine CFP1 is important for various aspects of oocyte development and zygotic genome activation (38,39). Consistent with a role in embryonic transcription, *Cfp1^−/-^*murine embryonic stem (ES) cells fail to differentiate (40–42). Intriguingly, like the nematode protein, CFP1 is temporally repressed in maturing murine oocytes and early embryos (38,39). Thus, the transient repression of CFP-1/CFP1 could be a conserved phenomenon important for the epigenetic reprogramming underlying the switch from germ- to embryonic transcription.

In other models, so-called pioneering transcription factors (Zelda in flies, Pou5f3, Sox19b and Nanog in fish, DUX and NFY in mice, and OCT4 in humans) play critical roles in activating the embryonic genome by increasing chromatin accessibility to other transcription factors (43). Among them, the fly Zelda and human *OCT4* were shown to be post-transcriptionally regulated. Zelda is the major activator of the zygotic genome and its expression in fly embryos is translationally repressed by the LIN-41 ortholog BRAT (44,45). As for OCT4, it activates transcription in the mouse embryos at the 2-cell stage when the zygotic transcription begins (46). In human embryonic stem cells and germinal vesicle-stage of pig oocytes, the *OCT4* mRNA associates with another RBP, DND1 (47,48). This association could be functionally relevant for the germline, as OCT4 expression is post-transcriptionally downregulated in the male germ cells where, analogous to LIN-41, DND1 maintains germline identity, preventing the onset of testicular teratomas (49,50). Also, similar to LIN-41 and BRAT (37), DND1-mediated translational repression involves the recruitment of CCR4–NOT deadenylase (36). Thus, while the general strategy of controlling the transcriptional reprograming during OET by posttranscriptional ‘roadblocks’ appears to be conserved (51), the mRNA targets may differ. Zelda is not conserved outside insects, and the nematode homologs of OCT4 and SOX2 are required for a transdifferentiation event in the soma, where the rectal epithelial cell Y transdifferentiates into PDA neurons (52). Perplexingly, however, these factors appear to have no role in the transcriptional reprogramming during OET, nor were their functional equivalents identified so far. In nematode embryos, histone acetyltransferases (HATs) promote most somatic differentiation programs likely by antagonizing histone deacetylase activities (53). In murine embryos, overexpression of a dominant-negative form of HDAC1/2 leads to a developmental arrest at the two-cell stage (54). In these embryos, 64% of the downregulated genes are EEGs and the authors proposed that HDAC activity is critical for the activation of the zygotic genome by creating correct transcription-active and -repressive states at the chromatin level. These examples stress the importance of epigenetic reprogramming for embryonic differentiation. While it remains possible that nematodes use a yet-to-be-found pioneering factor, our findings suggest that chromatin modifications mediated by CFP-1 and possibly other chromatin interacting proteins could play the main role in the switch from the germline-specific to embryonic transcription. In this scenario, transient repression and subsequent re-expression of CFP-1 could contribute to the erasure of germline-specific chromatin states and the subsequent establishment of a chromatin environment compatible with embryonic differentiation.

## Supplementary Material

gkad729_Supplemental_File

## Data Availability

The data underlying this article are available in the article and in its online supplementary material.
