# Supplementary material for: Post-transcriptional repression of CFP-1 expands the regulatory repertoire of LIN-41/TRIM71
Source: Nucleic Acids Res. 2023 Sep 6;51(19):10668–80. doi: 10.1093/nar/gkad729 (PMC10602926; doi:10.1093/nar/gkad729)
Supplement: gkad729_Supplemental_File [file gkad729_supplemental_file.pdf]

Post-transcriptional repression of CFP-1 expands the regulatory repertoire of LIN-41/TRIM71

Pooja Kumari[1], Lars Harald Thuestad[1], Rafal Ciosk[1, #]

[1] Department of Biosciences, University of Oslo, Oslo 0316, Norway

[#] To whom correspondence should be addressed. Tel: +47 22859070; Email: rafal.ciosk@ibv.uio.no

Supplementary Figures

Supplementary Tables

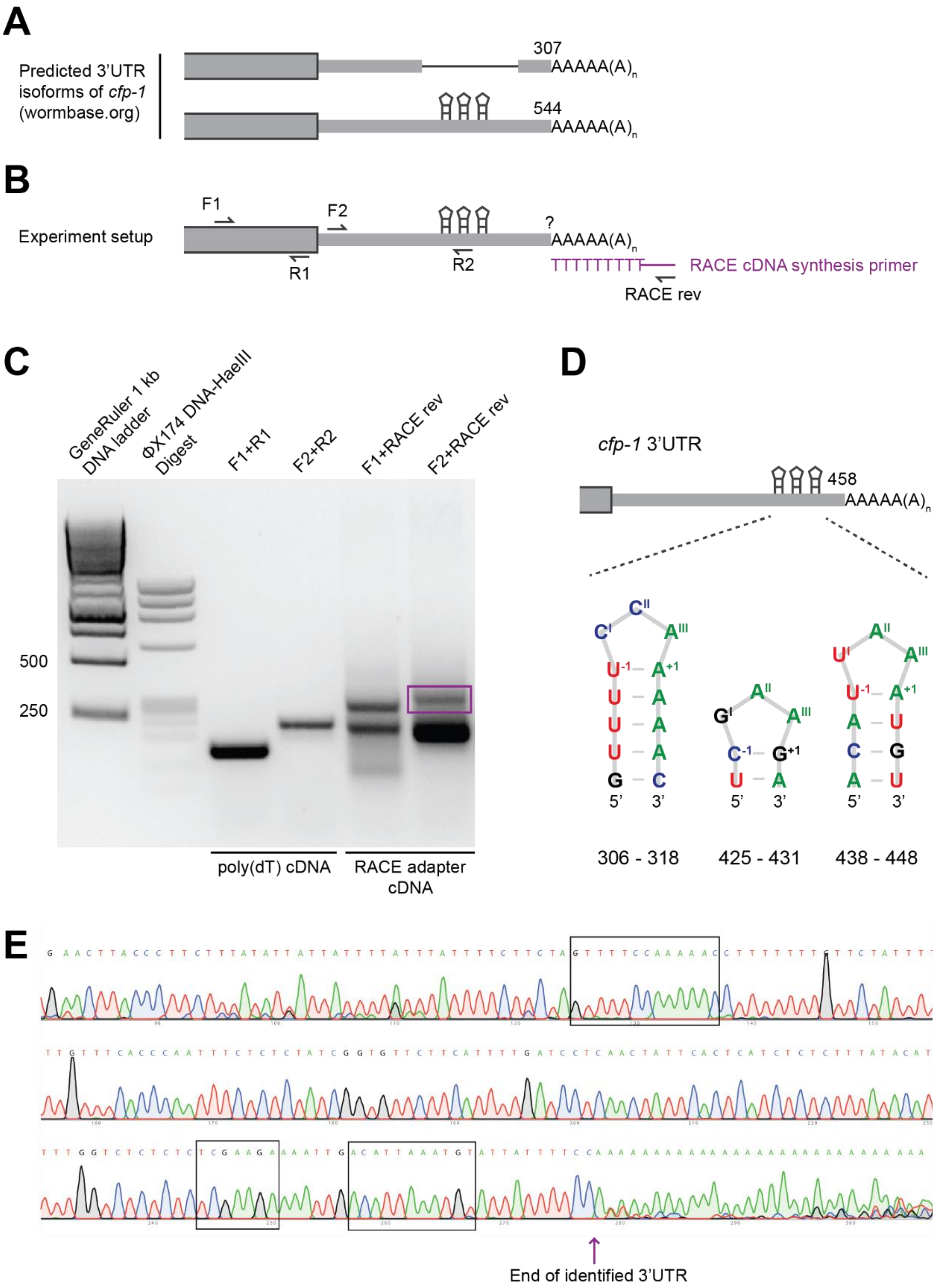
A
Predicted 3'UTR isoforms of cfp-1 (wormbase.org)
307
AAAAA(A)n
544
AAAAA(A)n
B
Experiment setup
F1
F2
R1
R2
?
AAAAA(A)n
TTTTTTTTT
RACE cDNA synthesis primer
RACE rev
C
GeneRuler 1 kb DNA ladder
ΦX174 DNA-HaeIII Digest
F1+R1
F2+R2
F1+RACE rev
F2+RACE rev
500
250
poly(dT) cDNA
RACE adapter cDNA
D
cfp-1 3'UTR
458
AAAAA(A)n
306 - 318
425 - 431
438 - 448
E
End of identified 3'UTR

Figure S1 - RACE assay to determine the 3'UTR of *cfp-1.*

A. Graphic representation of annotated *cfp-1* 3'UTR isoforms (WBGene00009924#0-9f1-10), where the shorter 3'UTR isoform (307 bp) does not contain predicted LREs and the longer 3'UTR isoform (544 bp) has three.

B. Schematic illustrating the positions of primers used for the PCR reactions in C. The RACE adapter primer used for cDNA synthesis is shown in purple.

C Image of electrophoresis gel used to separate the RACE PCR products. The purple box outlines the band that corresponds to identified 3'UTR isoform. PCR reactions were also performed with cDNA synthesized with poly(dT) primers to test the RNA quality and primers. The other PCR bands in the RACE lanes corresponded to bacterial genes. The 3' UTR of *cfp-1* is AT-rich and so are the primers which could be degenerate hence resulting in false priming of bacterial genes.

D. Schematic of the *cfp-1* 3'UTR isoform determined by RACE, with the length of 458 nt after which we detected the polyA tail. The sequences and positions of the three predicted LREs are shown.

E. Section of sequence chromatogram showing the three LREs (boxed) and the endpoint of the determined 3'UTR and the start of the poly(A)-tail which is earlier as compared to the annotated site.

aauuucccuauuuuuuuuaguuuuuuccucaaaacuaucauauucuuucauuuccguggaauuucguaucuuuuuucca
aaucugaacaacauuuuuuucuguuugaauaggugucguccugaaauauggcggagagagagagagugaagagacgcag
acacucuaacgacacguugcguauauuacacgaagcacuggcuuuuucauucguuguucuucaaaauuuuuaugauuuu
uaucuuuugaacaaaaaacaaauugaacuuacccuucuuuauauuauuauuuuauuuauuuucuucuaguuu**a**ccaaaa
accuuuuuuuguucuauuuuuuguuucacccaauuucucucuaucgguguucuucauuuugauccucaacuauucacuc
aucucucuuuauacauuuuggucucucucu**g**gaagaaaauugaca**a**uaaauguauuauuuuccauuuuccgaauuuuuc
aacaaaaaacuuuuauuccaaaaaaaauccuuaaaaaauuuaaaaaaaaacaccaaaaaaauuuuucaga

Figure S2 – Sequence of mutant *cfp-1* 3’UTR

The three bases in red are the point mutations used to disrupt the 3 LREs.

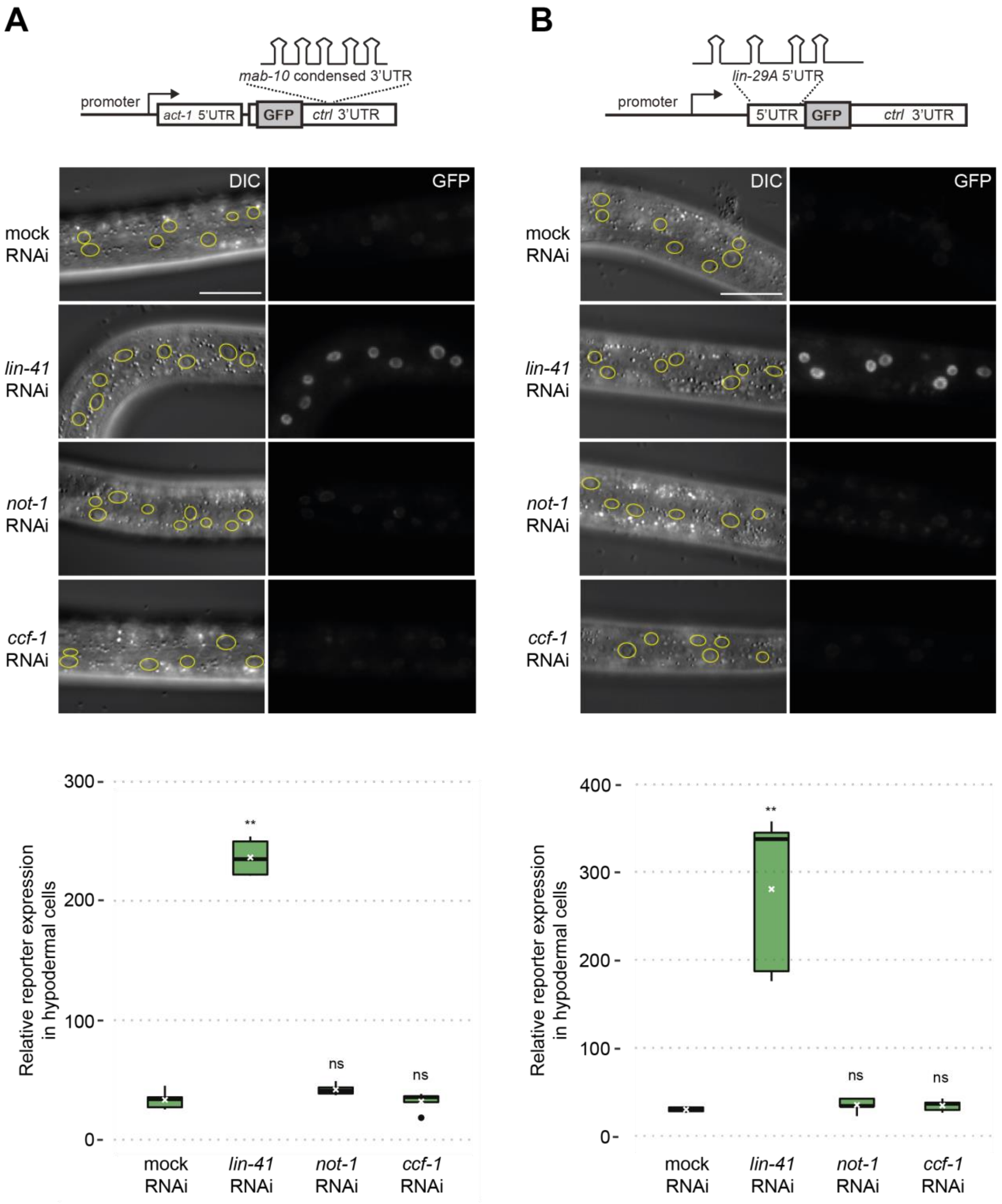

Figure S3 - The CCR4-NOT deadenylase complex is not required for the repression of somatic LRE reporters.

A. Top: Schematic of a somatic 3' UTR LRE reporter, wherein GFP is expressed under the control of the *lin29A* promoter, control *act-1* 5'UTR and a synthetic 3'UTR containing five LREs from the somatic mRNA target (*mab-10*). Middle: Micrographs of early L3-stage *C. elegans* larvae, treated with either *lin-41*, *not-1,*

ccf-1 or mock RNAi, showing reporter GFP in hypodermal cells (yellow circles demarcate cells), expressed from the reporter constructs illustrated above. Scale bar = 25 µm. Bottom: Quantification of pixel intensity illustrating the change in reporter GFP fluorescence, calculated as difference between the hypodermal cells and background fluorescence. Mean values are marked by white crosses. The p-value was calculated using the unpaired two-sample Wilcoxon test. ** indicates p value < 0.05.

B Top: Schematic of a somatic 5' UTR LRE reporter, wherein GFP is expressed under the control of the *dpy-30* promoter and an unregulated *unc-54* 3' UTR. The 5'UTR is from the somatic target *lin-29A* containing 4 LREs. Middle: Micrographs of early L3-stage *C. elegans* larvae, treated with either *lin-41*, *not-1, ccf-1* or mock RNAi, showing reporter GFP in hypodermal cells (yellow circles demarcate cells), expressed from the reporter constructs illustrated above. Scale bar = 25 µm. Bottom: Quantification of pixel intensity illustrating the change in reporter GFP fluorescence, calculated as difference between the hypodermal cells and background fluorescence. Mean values are marked by white crosses. The p-value was calculated using the unpaired two-sample Wilcoxon test. ** indicates p value < 0.05.

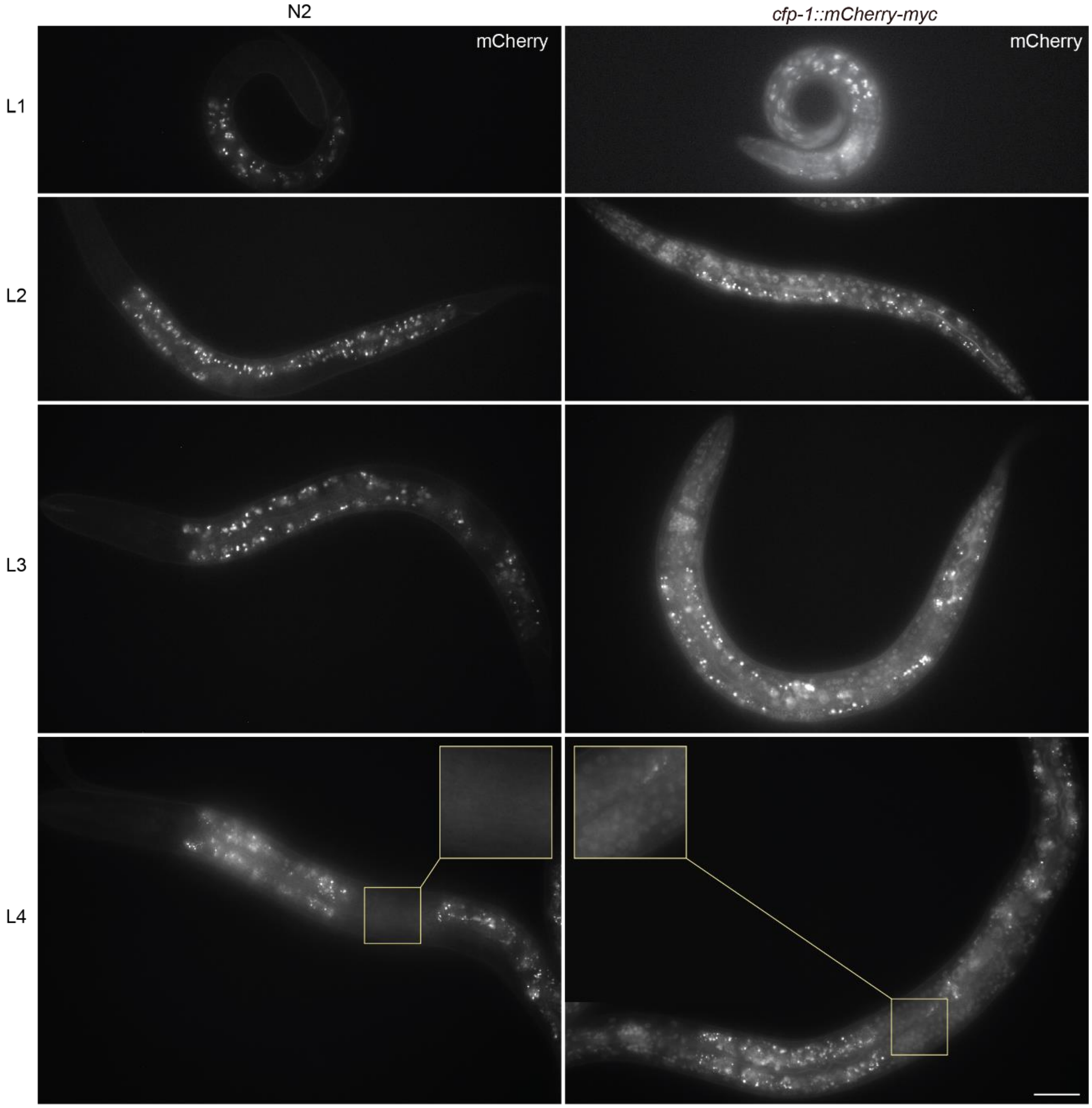

Figure S4 – CFP-1 expression in larvae.

Fluorescence micrographs of L1-L4 larvae from the wild-type strain N2 and *cfp-1::mCherry-myc* strain expressing CFP-1 fused to mCherry and myc. Boxed areas and the corresponding two-fold magnifications shown partial views of the L4 gonads. CFP-1::mCherry-myc was expressed in both germline and somatic cells. Scale bar is 25 µm.

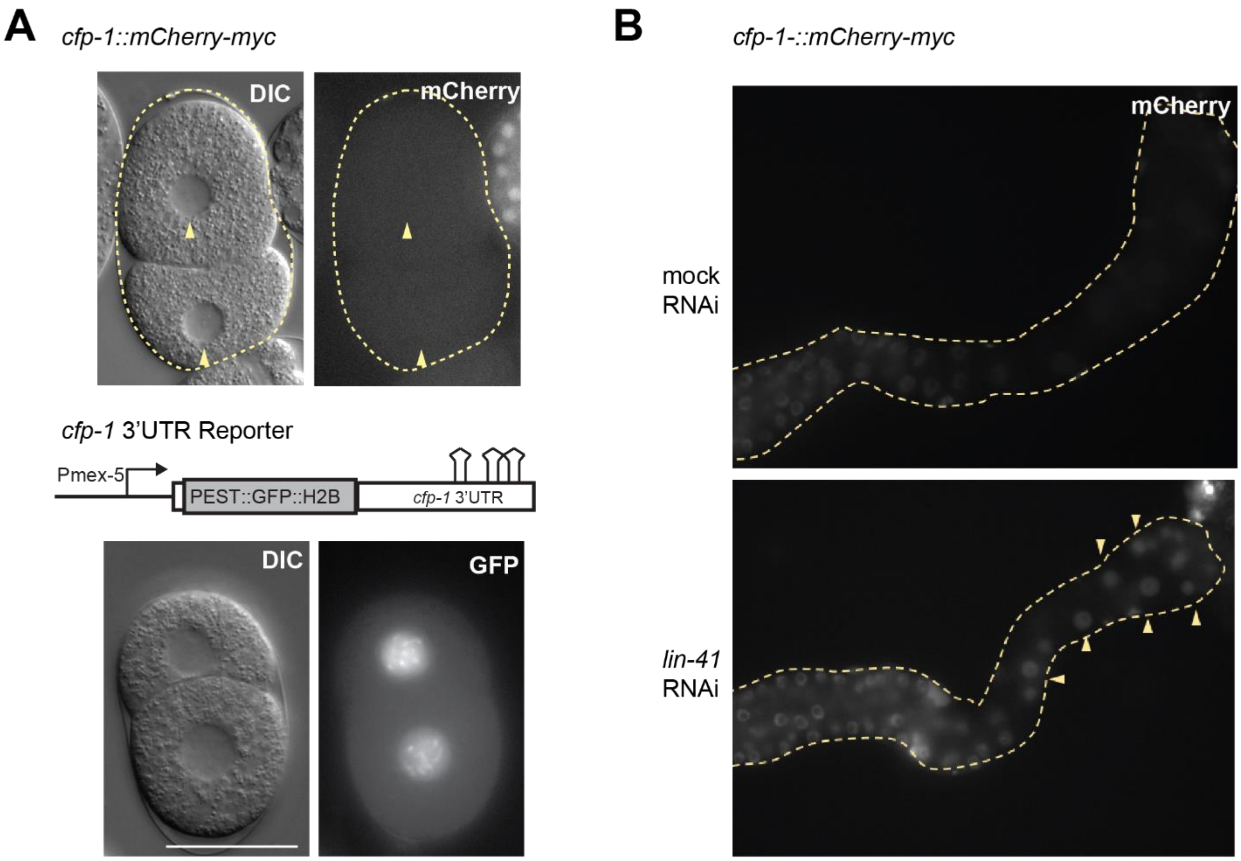

Figure S5 – Expression of the CFP-1 fusion protein and the *cfp-1* 3'UTR GFP reporter.

A. Top: DIC and fluorescence micrographs of a 2-cell stage embryo from the strain *cfp-1::mCherry-myc*. Bottom: DIC and fluorescence micrographs of a 2-cell stage embryo from the *cfp-1* 3'UTR GFP reporter strain. Scale bar is 25 µm.

B. Fluorescence micrographs of gonads (outlined) dissected from the *cfp-1::mCherry-myc* strain subjected to mock or *lin-41* RNAi. The arrowheads mark the nuclei of oocyte-like cells in the *lin-41* RNAi gonad expressing CFP-1::mCherry-Myc.

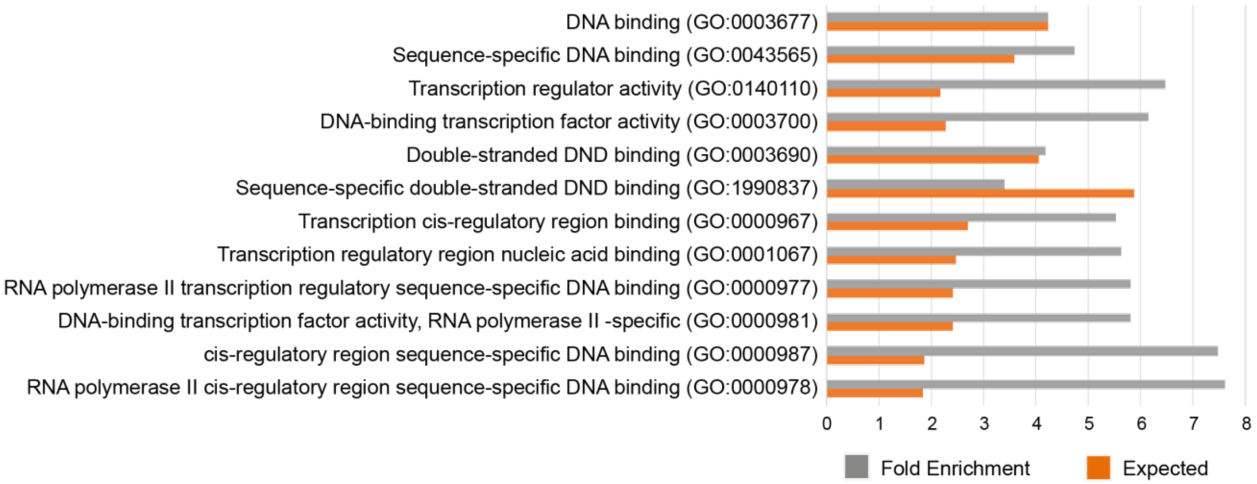

Figure S6 – Gene ontology enrichment analysis on EEGs downregulated in *cfp-1* mutants.

GO analysis for molecular function on the overlapping genes between EEGs and genes downregulated in *cfp-1(tm6369)* mutants (Figure 6A, left panel) using the gene ontology resource (http://geneontology.org/). Determined fold enrichments are plotted against expected fold enrichments for various GO molecular functions.

Identifier	Genotype	Source
N2	Wild-type	CGC
RAF1894	rrrSi447[Pmex-5::GFP::H2B::PEST::unc-54+mab-10 (500bp) 3'UTR; unc-119(+)] II; unc-119(ed3) III	This study
RAF1180	*lin-41(rrr3)/unc-29(e1072), lin-11(h1281) (I); rrrSi199 (II); rrrSi198 (IV)*	Tocchini et al., 2014
RAF1163	*lin-41(rrr3)*	Tocchini et al., 2014
RAF2216 (PHX5469)	*syb1s5469*[(P*mex-5*::*gfp*::*H2B*::*PEST*::*cfp-1* 3'UTR) **II**]; *unc-119*(ed3) III	Suny biotech
RAF2240 (PHX5817)	*sybIs5817*[(P*mex-5*::*gfp*::*H2B*::*PEST*::*cfp-1* 3'UTR(LRE mutant)) **II**];*unc-119*(ed3) III	Suny biotech
RAF2190 (PHX3876)	*cfp-1*(*syb3876*[*cfp-1*::*mCherry*::*myc*])IV	Suny biotech
RAF2210	*lin-41*(*tn1487ts*[D1125N])I; *cfp-1*(*syb3876*[*cfp-1*::*mCherry*::*myc*])IV	Cross between *lin-41(tn1487ts)* from Spike et al, 2014 and RAF2190
RAF2011	*EG6699, xeSi318[*P*lin-29A(act-1* 5'UTR exon*)::gfp(pest)/h2b::unc-54 3'UTR(mab-10* fused SL*), unc-119*(+)] II	Kumari et al., 2018
RAF2007	*EG6699, xeSi147[*P*dpy-30::lin-29A* 5'UTR exon 1*::gfp(pest)/h2b::unc-54* 3'UTR*, unc-119*(+)] II	Aeschimann et al., 2017

Table S1 - *C. elegans* strains

The strain ID used in the Ciosk lab, corresponding genotype and source are listed.

RNA polymerase II cis-regulatory region sequence-specific DNA binding (GO:0000978)

Primer	Sequence
act-1 FW	AGGACTTGTACGCCAACACT
act-1 RV	TGGAGAGGGAAGCGAGGATA
cfp-1 FW	ACAAGTCCCAATCCACGTCA
cfp-1 RV	CCTCCTCCTTCTGTACAGCC
set-21 FW	GTACACGGACGTCCTGAAAG
set-21 RV	GCCAAGTCGCGATCTTCTTT
jmjd-2 FW	CGGAACAGGCTGAAAACGAA
jmjd-2 RV	AAGAGGCGCAGAGATCACAT
lsd-1 FW	AACCGAACCCCAACACTTTG
lsd-1 RV	ACAAGTGTTCGCCAAGTCAG
spn-4 FW	GTCCGTTTAACCTACCTCGC
spn-4 RV	GCACTTCATTTGACTTCGAGC
orc-1 FW	TACTCTTTCCTGCTCGCCTT
orc-1 RV	TTAGGGGAGAATGTGCGAGG
F01F1.15 FW	CACCTCTACCTGCTGCTCC
F01F1.15 RV	AGCACAATTTCCAGATCCCG
CC8.2 FW	TCTCCACGAAAGGCCCAAG
CC8.2 RV	GAGCCGAGGACACGTAGG
F14H3.4 FW	CGAAGAAAAGGAAGCAGGTGA
F14H3.4 RV	GGCCTTCCTCATCATCCTCA
mis-12 FW	AGAAAATTCGACAGCTCCGC
mis-12 RV	CATTCGTGTTGGGCTATCGG
mex-3 FW	CATGGTCGTCGATGGGATTG
mex-3 RV	ATGAAGTCGGCGAGGCAG
cfp-1 F1	ACAAGTCCCAATCCACGTCA
cfp-1 F2	GCAGACACTCTAACGACACG
cfp-1 R1	CCTCCTCCTTCTGTACAGCC
cfp-1 R2	AGGATCAAAATGAAGAACACCGA

Table S2 – Primer sequences
